# Histopathological Assessment of Cellular Heterogeneity in Pediatric Ependymomas

**DOI:** 10.3390/diagnostics15243144

**Published:** 2025-12-10

**Authors:** Murad Alturkustani

**Affiliations:** 1 Department of Pathology, Faculty of Medicine, King Abdulaziz University, Jeddah 21589, Saudi Arabia; alturkustani.murad@gmail.com; 2 Department of Pathology and Laboratory Medicine, Western University, London, ON N6A 5C1, Canada

**Keywords:** pediatric ependymoma, histopathology, GFAP, EMA, cellular heterogeneity, infiltrative astrocytic-like cells, diagnostic classification, prognostic markers

## Abstract

**Background/Objectives:** Ependymomas are central nervous system (CNS) tumors with marked biological and clinical heterogeneity, particularly in pediatric populations. While the 2021 World Health Organization (WHO) classification emphasizes molecular subgroups—posterior fossa type A (PFA) and B (PFB), supratentorial *ZFTA* fusion-positive (ST-ZFTA), and *YAP1* fusion-positive (ST-YAP)— routine diagnosis is still based on histology and immunohistochemistry (IHC). Recent single-cell RNA sequencing and spatial transcriptomic studies have revealed distinct tumor cell populations, including ependymal-like, astroglial-like, progenitor-like, and stress-associated states. However, a major unresolved issue is whether such heterogeneity can be appreciated and interpreted on conventional pathology slides. **Methods:** This study examined ependymomas from the Children’s Brain Tumor Network (CBTN), with hematoxylin and eosin (H&E) and IHC for glial fibrillary acidic protein (GFAP) and epithelial membrane antigen (EMA). Tumor regions were stratified into high-cellularity and low-cellularity regions, and staining patterns were correlated with known cellular features from the prior literature. **Results:** Low-cellularity zones exhibit strong fibrillary GFAP, resembling astroglial or subependymal differentiation. In contrast, high-cellularity zones more often demonstrate variable EMA patterns and GFAP/EMA-negative compartments, consistent with undifferentiated progenitor-like populations. Perinecrotic areas showed increased GFAP and EMA, possibly reflecting stress-associated cellular states and mesenchymal differentiation. Comparisons between PFA and ST-ZFTA tumors revealed that ST-ZFTA ependymomas were significantly more likely to be hypercellular, with a higher frequency of diffuse EMA expression. In contrast, PFA tumors displayed broader variability with stronger GFAP perinuclear staining. **Conclusions:** These findings support the concept that conventional histology can capture relevant heterogeneity and may complement molecular studies. The recognition of such features may help refine histopathological assessment and provide practical prognostic insights, particularly in resource-limited settings where molecular testing is not universally available.

## 1. Introduction

Ependymomas are glial tumors that arise throughout the central nervous system (CNS). The diagnosis and classification of ependymomas have relied on histological features such as cellularity, mitotic index, microvascular proliferation, and necrosis [1]. Although such features provide some prognostic information, clinical outcomes vary widely within histologically similar cases, highlighting the limitations of morphology alone [2].

Advances in molecular profiling, particularly DNA methylation and RNA sequencing, have revolutionized the classification of ependymomas. The current WHO classification recognizes several molecularly defined entities: supratentorial *ZFTA* fusion-positive (ST-ZFTA), supratentorial *YAP1* fusion-positive (ST-YAP), posterior fossa type A (PFA), posterior fossa type B (PFB), and rare subtypes such as spinal MYCN-amplified ependymoma [3]. These molecular categories demonstrate stronger correlations with prognosis than traditional histopathology: PFA tumors in children show poor survival, while PFB tumors and *YAP1*-driven supratentorial ependymomas are associated with more favorable outcomes [4].

Recent single-cell transcriptomic studies provide important clues. Gillen et al. identified undifferentiated progenitor-like cells, ependymal-like populations, astroglial-like populations, and hypoxia-associated mesenchymal states within PFA tumors [5]. In parallel, Gojo et al. described neural stem-like, neuronal precursor-like, glial progenitor-like, and ependymal/astroependymal-like tumor cell populations [6]. Despite differences in terminology, both studies converge on the principle that pediatric ependymomas contain multiple tumor-intrinsic programs that correlate with the prognosis.

The prognosis of ependymoma varies according to the proportion of cellular density and cell types, which is promising for further refining prognostic factors and enhancing our understanding of the disease. However, a significant gap in current knowledge is how to translate these molecular insights into routine histopathological assessment, particularly in resource-limited settings. Thus, identifying histological and immunohistochemical markers that confirm the possibility of identifying this cellular heterogeneity could pave the way for better refining the histological features in grading ependymoma.

The present study was designed to explore whether such heterogeneity is visible with conventional histology and IHC. Specifically, tumor regions were stratified by cellular density, and glial fibrillary acidic protein (GFAP) and epithelial membrane antigen (EMA) immunostaining were evaluated to determine whether histological and immunophenotypic differences could suggest the cellular states described in recent molecular work. This study aims to bridge the gap between molecular and morphological classification and to propose that routine pathology can, at least partially, capture the biologically meaningful heterogeneity of pediatric ependymomas.

## 2. Materials and Methods

Cases were obtained from the Children’s Brain Tumor Network (CBTN), a collaborative resource that integrates genomic and histological data from pediatric brain tumors. The CBTN database compiles anonymized and de-identified patient data from various contributing institutions, ensuring confidentiality. All data in this repository are collected strictly after receiving informed consent from the participants or their legal guardians, in strict compliance with ethical standards and guidelines.

All patients in this analysis were younger than 19 years at diagnosis and thus considered pediatric. A total of 97 methylation-classified ependymoma cases were initially screened in the OpenPBTA dataset (last accessed on October 2025). Cases were eligible if they met all of the following criteria: (1) classification as ependymoma by the Heidelberg brain tumor methylation classifier v12.5 with an appropriate methylation class score; (2) availability of digital slides stained with hematoxylin and eosin (H&E), glial fibrillary acidic protein (GFAP), and epithelial membrane antigen (EMA); and (3) sufficient slide quality to allow assessment of cellularity and immunostaining patterns. Cases that lacked complete GFAP and/or EMA immunostaining, had inadequate image quality, or had low-confidence methylation scores were excluded. Nineteen cases met all inclusion criteria and were included in the final analysis. These comprised 10 posterior fossa type A (PFA), eight supratentorial *ZFTA* fusion-positive (ST-ZFTA), and one posterior fossa type B (PFB) ependymoma.

All available digital slides were centrally reviewed by a single board-certified neuropathologist and pediatric pathologist (M.A.), using the unified scoring criteria described below. No additional central review panel was used.

**Figure 1:**
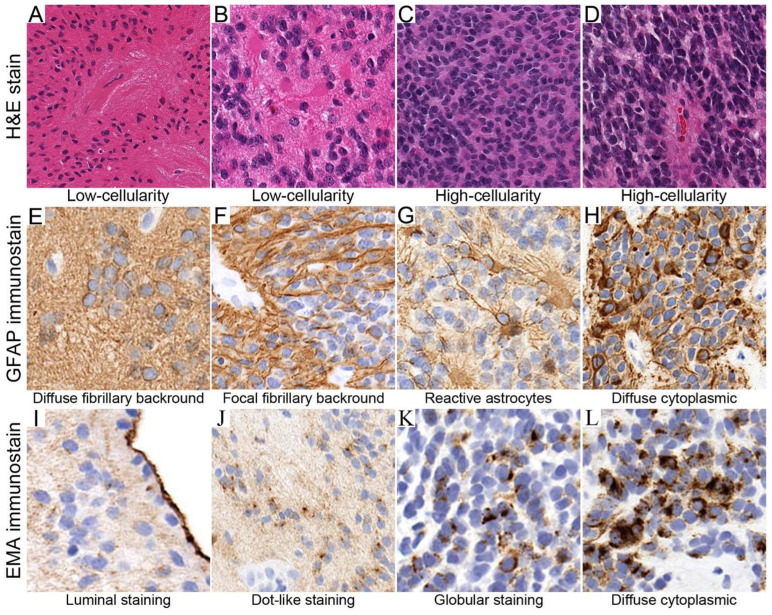
Definition of histological and immunohistochemical features used for analysis. (**A**–**D**) Tumor cellularity assessment on H&E sections: regions with less than 50% were considered low-cellularity, whereas those with more than 50% tumor nuclei were classified as high-cellularity. (**E**–**H**) GFAP immunostaining patterns showing diffuse fibrillary background, perinuclear cytoplasmic rim staining (**E**), and cytoplasmic processes, which were further classified as reactive-appearing (thin, delicate processes) (**G**) or neoplastic-appearing (coarse, irregular processes) (**H**). (**I**–**L**) EMA immunostaining patterns demonstrating luminal, dot-like, granular/globular, and diffuse cytoplasmic.

Tumor cellularity was assessed on H&E sections by estimating the proportion of tumor nuclei relative to background. Regions with more than 50% tumor nuclei were defined as high-cellularity, and those with less than 50% were low-cellularity (Figure 1A–D). This threshold mirrors the classification used by Gödicke et al., who showed that cell-dense versus cell-poor regions correlate with molecular changes and prognosis [7].

Immunohistochemical staining for GFAP and EMA was performed on formalin-fixed, paraffin-embedded (FFPE) tissue sections. Staining intensity and distribution for both immunostains were scored semi-quantitatively as follows: (0) No staining, (1+ or weak) staining in <10% of tumor cells, (2+ or moderate) staining in 10–50% of tumor cells, and (3+ or strong) staining in >50% of tumor cells.

GFAP staining was assessed for intensity and distribution. Staining was categorized as diffuse fibrillary (background processes involving more than 50% of the region), focal fibrillary (less than 50%), perinuclear cytoplasmic rim (restricted to cytoplasm around the nucleus with no processes), or cytoplasmic processes. The latter was further divided into reactive-appearing (thin and delicate, similar to reactive astrocytes elsewhere) and neoplastic-appearing (coarse and irregular, similar to neoplastic astrocytes in astrocytoma) (Figure 1E–H).

EMA staining was categorized as dot-like paranuclear, granular cytoplasmic, globular, diffuse cytoplasmic, luminal, and ring-like staining. Granular staining was defined as many small dots, while globular staining was defined as fewer, larger globules filling the cytoplasm, and both were considered under the same category (Figure 1I–L).

Cellularity proportions were compared between groups using Fisher’s exact test due to small sample size and zero counts in some subcategories. Statistical significance was set at *p* < 0.05.

## 3. Results

The study cohort included 19 pediatric patients with ependymomas, comprising 13 males and six females, with ages ranging from 4 months to 18 years. Molecular classification identified 10 PFA cases, 8 ST-ZFTA cases, and a single PFB case. Overall survival (OS) ranged from 2 to 95 months, while progression-free survival (PFS) ranged from 1 to 92 months. For PFA, the median OS was 42 months, and the median PFS was 22 months, whereas ST-ZFTA tumors had a median OS of 55 months, and a median PFS of 37 months. The majority of patients remained alive at the last follow-up (14 patients), while five had died. The single PFB case was alive at 2 months of follow-up. These findings underscore the variability in clinical courses and highlight the potential impact of molecular subtype on survival outcomes. Detailed pathological findings are summarized in Table 1.

**Table 1:** Clinical and pathological findings in pediatric ependymomas.

Case Number	Age	Sex	Overall Survival in Months	Progression- Free Survival in Months	Status	MC (Score)	Cell Density	Percentage	GFAP				EMA		
									Fibrillary Background	Perinuclear Rim	Neoplastic Looking	Reactive Looking	Dot-like	Granular/ Globular	Diffuse Cytoplasmic
1	1 year	M	70	4	Alive	PFA (0.99)	Low-cellular	70	3	0	0	2	2	2	0
							High-cellular	30	2	1	0	3	2	2	0
2	1 year	M	62	62	Alive	PFA (0.95)	Low-cellular	NA	NA	NA	NA	NA	NA	NA	NA
							High-cellular	100	3	3	2	0	3	3	2
3	11 years	M	30	3	Dead	PFA (0.96)	Low-cellular	NA	NA	NA	NA	NA	NA	NA	0
							High-cellular	100	2	2	2	0	1	1	0
4	4 months	F	2	2	Alive	PFA (0.99)	Low-cellular	20	3	2	0	0	3	2	0
							High-cellular	80	3	2	0	0	1	0	0
5	3 years	M	95	35	Alive	PFA (0.99)	Low-cellular	10	3	3	3	0	3	3	2
							High-cellular	90	2	2	2	2	3	3	0
6	17 years	M	4	4	Alive	PFA (0.96)	Low-cellular	80	3	2	0	0	2	3	2
							High-cellular	20	3	2	0	0	2	3	2
7	11 years	M	60	60	Alive	PFA (0.99)	Low-cellular	80	3	3	0	0	1	1	1
							High-cellular	20	3	3	0	0	1	1	1
8	3 years	M	20	3	Dead	PFA (0.99)	Low-cellular	40	3	0	0	0	2	2	0
							High-cellular	60	2	0	0	2	2	2	2
9	11 months	F	13	13	Alive	PFA (0.85)	Low-cellular	80	3	2	0	2	2	3	3
							High-cellular	20	2	2	0	2	2	2	0
10	7 years	F	55	55	Alive	PFA (0.99)	Low-cellular	10	3	2	0	0	2	2	0
							High-cellular	90	2	2	3	0	3	3	3
11	4 years	F	31	1	Dead	SF-ZFTA (0.99)	Low-cellular	30 *	NA	NA	NA	NA	NA	NA	NA
							High-cellular	70	0	0	0	0	1	3	3
12	4 years	F	54	54	Alive	SF-ZFTA (0.99)	Low-cellular	30	3	0	0	2	2	0	0
							High-cellular	70	0	0	2	3	2	2	2
13	4 years	F	67	20	Dead	SF-ZFTA (0.99)	Low-cellular	NA	NA	NA	NA	NA	NA	NA	NA
							High-cellular	100	0	0	3	0	1	2	2
14	1 year	M	92	92	Alive	SF-ZFTA (0.99)	Low-cellular	30	3	3	1	1	2	2	2
							High-cellular	70	2	0	1	2	2	3	3
15	6 years	M	56	56	Alive	SF-ZFTA (0.99)	Low-cellular	NA	NA	NA	NA	NA	NA	NA	NA
							High-cellular	100	3	1	1	3	3	3	3
16	16 years	M	7	4	Dead	SF-ZFTA (0.99)	Low-cellular	10	3	3	2	0	2	2	0
							High-cellular	90	2	0	0	2	3	3	3
17	12 years	M	55	55	Alive	SF-ZFTA (0.99)	Low-cellular	NA	NA	NA	NA	NA	NA	NA	NA
							High-cellular	100	2	0	2	2	3	3	2
18	8 months	M	9	9	Alive	SF-ZFTA (0.77)	Low-cellular	NA	NA	NA	NA	NA	NA	NA	NA
							High-cellular	100	2	2	2	1	3	3	2
19	18 years	M	61	2	Alive	PFB (0.98)	Low-cellular	10	3	2	0	1	3	3	2
							High-cellular	90	2	2	1	2	3	2	1

* The EMA stain in the low-cellular component was not interpretable due to technical issues. Staining scores: 1 = <5%, 2 = 5–50%, 3 = >50%. MC: methylation class; NA: not applicable.

**Figure 2:**
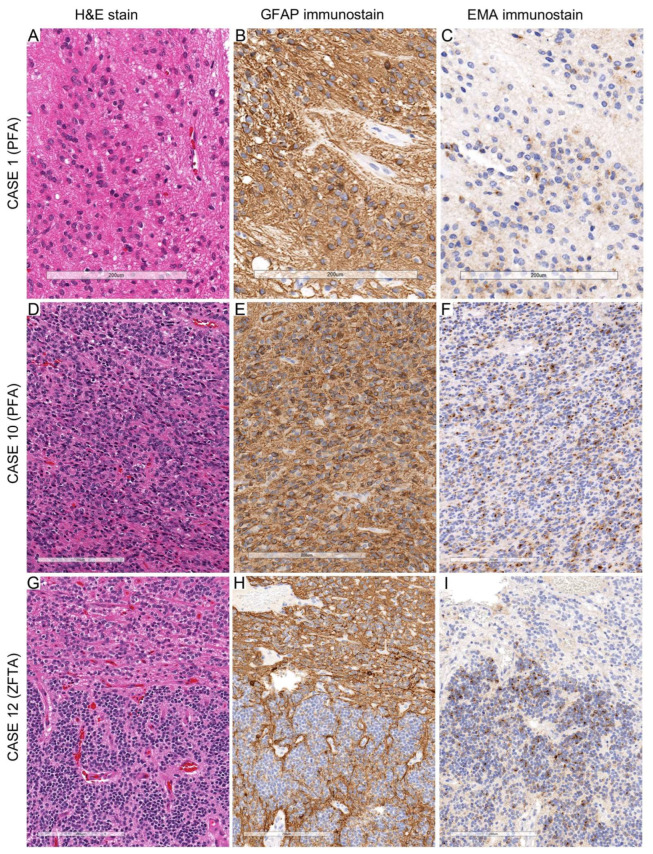
Features of low- and high-cellularity regions in ependymomas. (**A**–**C**) Case 1 (PFA) showing a low-cellularity region with a diffuse fibrillary background and prominent perinuclear rim staining for GFAP (**B**), and dot-like EMA positivity (**C**). (**D**–**F**) Case 10 (PFA) demonstrating a high-cellularity region with strong GFAP perinuclear rim staining within dense tumor areas (**E**) and sparse dot-like EMA reactivity (**F**). (**G**–**I**) Case 12 (ZFTA) displaying adjacent low-cellularity (upper half) and high-cellularity (lower half) regions. The low-cellularity area shows a diffuse GFAP-positive fibrillary background with minimal perinuclear rim staining, while the high-cellularity region shows focal background staining and limited GFAP expression in neoplastic cells (**H**). EMA immunostaining highlights predominantly globular cytoplasmic positivity in the high-cellularity area, with only scattered dot-like staining in the low-cellularity region (**I**). Scale bars: 200 µm (**A**–**I**).

Overall, ependymomas exhibited regions with variable cellularity and distinct immunohistochemical profiles. Low-cellularity regions were generally uniform across all subtypes, characterized by a prominent fibrillary GFAP background and diffuse GFAP staining, while EMA staining predominantly exhibited a dot-like pattern (Figure 2A–C). In contrast, high-cellularity regions displayed marked heterogeneity, with GFAP staining showing variable intensity and occasional absence, and EMA demonstrating dot-like, granular, and diffuse cytoplasmic patterns that differed between subtypes (Figure 2D–F). This pattern highlights that while low-cellularity areas are relatively consistent, high-cellularity regions reflect intra-tumoral heterogeneity and may provide insight into the underlying tumor biology.

Quantitative assessment of tumor cellularity revealed that the PFA group (10 cases) had a mean high-cellularity area of 61.0% and a mean low-cellularity area of 39.0%, with a standard deviation of 35.1%, reflecting considerable variability. High-cellularity areas ranged from 20% to 100% (median 70%), while low-cellularity areas ranged from 0% to 80% (median 30%). In contrast, ST-ZFTA tumors (8 cases) demonstrated a higher mean high-cellularity area of 87.5% and a lower mean low-cellularity area of 12.5%, with a smaller standard deviation of 14.9%, indicating more consistent cellularity. High-cellularity regions ranged from 70% to 100% (median 95%), and low-cellularity regions ranged from 0% to 30% (median 5%). The single PFB tumor exhibited a high-cellularity area of 90% and a low-cellularity area of 10%. Stratifying cases based on high-cellularity proportion (>50% vs. ≤50%) revealed that 60% of PFA tumors fell into the >50% group, whereas all ST-ZFTA tumors (100%) exceeded this threshold. Fisher’s exact test confirmed a significant difference between these subtypes (*p* = 0.044), indicating that ST-ZFTA tumors are more likely to exhibit high cellularity compared with PFA.

In the low-cellularity areas, all tumor types demonstrated a uniformly strong GFAP fibrillary background (Table 1). Perinuclear rim (PN) staining was more frequent and more substantial in PFA compared to ZFTA and PFB, suggesting it may be a supportive feature in distinguishing PFA. Reactive astrocyte-like staining was largely absent in PFA but showed a broader spectrum of expression in ZFTA, indicating greater heterogeneity in the latter. Neoplastic astrocyte-like staining was rarely seen in PFA and was generally weak in ZFTA and absent in PFB. For EMA, dot-like and cytoplasmic granular staining were standard in PFA and consistently strong in PFB, while ZFTA displayed weaker and less frequent patterns (Figure 2). Diffuse cytoplasmic EMA staining was variable in PFA, minimal in ZFTA, and intermediate in the single PFB case. Overall, the profile suggests that PFA tends to show stronger GFAP perinuclear staining and more robust EMA dot/granular positivity. In contrast, ZFTA demonstrates more variable GFAP reactivity and weaker EMA patterns. The single PFB case resembled PFA more closely in its staining profile.

In the high-cellularity regions, GFAP staining patterns showed greater variability compared with low-cellularity areas (Table 1). PFA cases frequently retained a strong fibrillary background, although a subset demonstrated reduced staining, whereas ZFTA showed a tendency toward having less fibrillary background overall. Perinuclear rim staining was a prominent feature in PFA, often scoring in the intermediate-to-strong range, while it was largely absent in ZFTA, with only occasional moderate positivity (Figure 2). EMA staining in high-cellularity areas highlighted more precise distinctions. Dot-like and globular cytoplasmic patterns were frequent in both groups, but they were more consistently strong in ZFTA compared with PFA, where staining was more variable. Diffuse cytoplasmic EMA staining was often weak or absent in PFA, whereas ZFTA cases frequently demonstrated moderate to strong diffuse positivity, marking a notable difference between the two entities. The single PFB case largely mirrored the PFA profile, with intermediate GFAP staining and EMA positivity. This diffuse cytoplasmic EMA staining was related to necrotic foci in high cellular regions.

**Figure 3:**
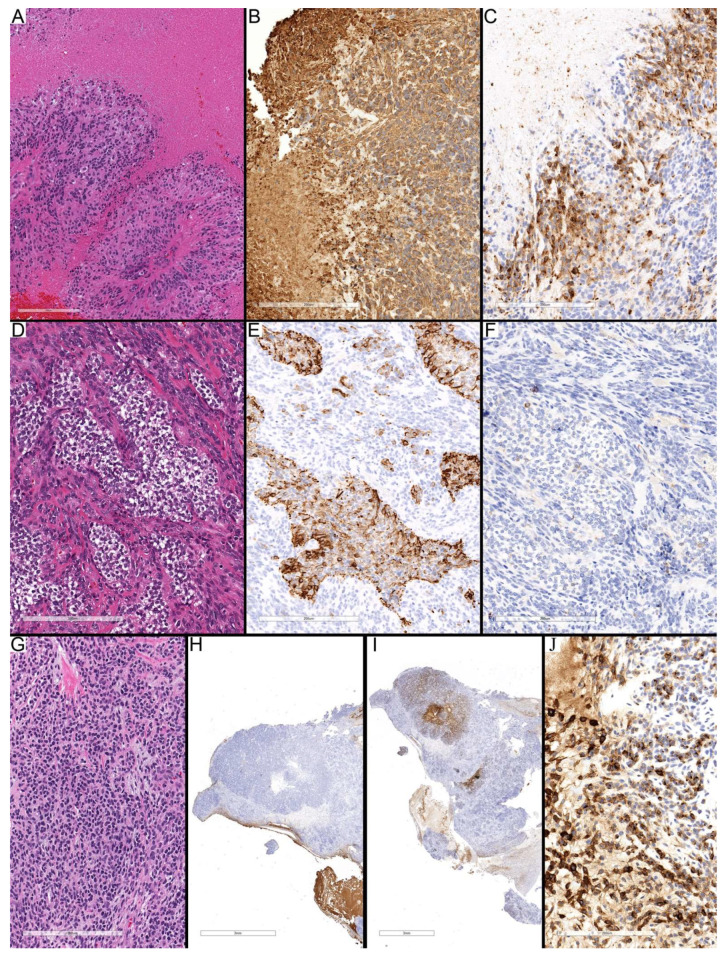
Different staining patterns in high-cellularity regions and around necrosis. (**A**–**C**) Case 2 (PFA) showing central necrosis surrounded by viable tumor cells. GFAP immunostaining (**B**) demonstrates similar staining intensity to non-necrotic regions, whereas EMA (**C**) shows diffuse cytoplasmic positivity around necrotic areas compared with dot-like and globular staining in viable regions away from necrosis. (**D**–**F**) Case 13 (ZFTA) illustrating compact areas of clear tumor cells with diffuse cytoplasmic GFAP staining (**E**), consistent with neoplastic-like reactivity, while the surrounding regions are less compact and show only focal GFAP positivity. EMA immunostaining (**F**) is negative in both the clear-cell and surrounding areas. (**G**–**J**) Case 11 (ZFTA) demonstrating compact epithelioid high-cellularity areas lacking GFAP expression (**H**). EMA immunostaining reveals variable globular and diffuse cytoplasmic positivity, particularly accentuated near the necrotic center (**I**,**J**). Scale bars: 200 µm (**A**–**G**,**J**), 3 mm (**H**,**I**).

Necrosis was identified in 4 of 10 PFA cases and in all 8 ZFTA cases, whereas it was absent in the single PFB case. The perinecrotic regions were characterized by increased EMA immunoreactivity forming broad geographic rims surrounding the necrotic centers (Figure 3A–C), observed in 2 of 4 necrotic PFA tumors and in 4 of 8 ZFTA tumors. Other EMA patterns, including luminal and ring-like staining, were noted; luminal EMA staining was present in 6 of 10 PFA cases but absent in ZFTA and PFB, while actual ring-like patterns were not readily identified, with most cases demonstrating globular cytoplasmic positivity.

The high-cellularity regions revealed heterogeneous cellular groups, including areas with diffuse GFAP cytoplasmic staining, while negative for EMA (Figure 3D–F), which may suggest neoplastic-appearing astrocytes (4 of 10 PFA and 4 of 8 ZFTA cases) morphologically. Other regions exhibited diffuse staining for EMA, whereas they were entirely negative for GFAP, particularly around the necrotic center (Figure 3G–J). The morphological appearance, location around the necrotic focus, and staining pattern suggest mesenchymal differentiation. Some zones were negative for both GFAP and EMA, representing undifferentiated or progenitor-like tumor cells, more pronounced in ST-ZFTA tumors. Taken together, these results suggest that cellularity and immunohistochemical patterns provide useful insight into tumor heterogeneity and may reflect subtype-related biological differences, with high-cellularity regions showing greater variability and more distinct subtype-associated features in this small cohort.

**Figure 4:**
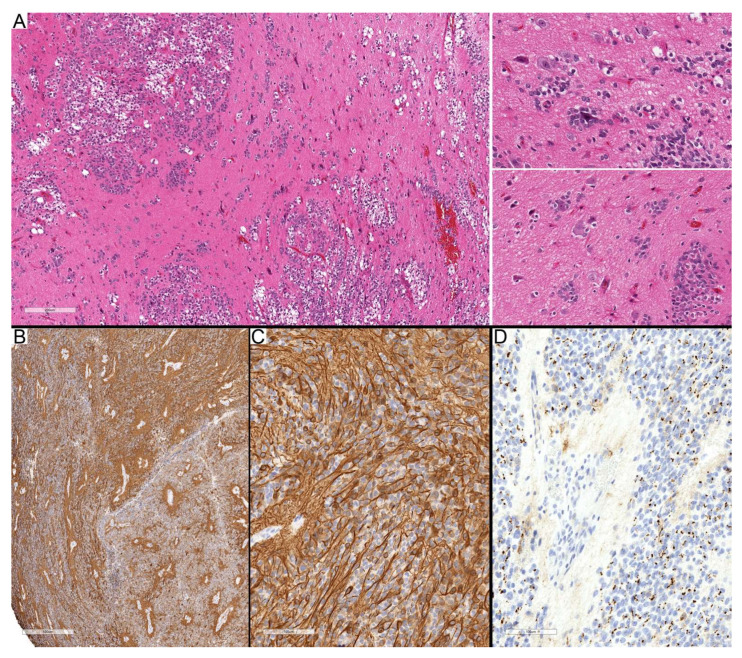
Infiltration in ZFTA ependymoma. (**A**) Case 18 shows infiltrative nests and individual tumor cells surrounding neurons, with higher-magnification (two panels on the right side) highlighting perineuronal infiltration by neoplastic cells. (**B**) Low-power GFAP immunostaining demonstrates a diffuse fibrillary background in low-cellularity regions (left upper part), while in high-cellularity areas (right lower part), GFAP reactivity is largely confined to perivascular zones. (**C**) Some GFAP-positive tumor cells display diffuse cytoplasmic staining with thick processes, features atypical of reactive astrocytes and more suggestive of neoplastic astrocytic-like differentiation. (**D**) EMA immunostaining reveals dot-like and granular cytoplasmic positivity in the tumor cells within the same regions. Scale bars: 200 µm (**A**), 200 µm (**B**), and 100 µm (**C**,**D**).

Notably, Case 18 demonstrated an unusual infiltrative growth pattern, with tumor cells forming infiltrative nests and perineuronal satellitosis resembling the Scherer structures seen in astrocytomas (Figure 4A). In the well-circumscribed regions of the ependymoma, the tumor exhibited the typical features of ST-ZFTA ependymoma, with GFAP staining showing a diffuse fibrillary background in low-cellularity areas (Figure 4B). In the high-cellularity regions, GFAP expression in the background was confined mainly to perivascular zones. At the same time, neoplastic cells displayed cytoplasmic GFAP staining with thick processes, suggestive of neoplastic astrocytic rather than reactive astrocytes (Figure 4C). EMA staining in these areas demonstrated dot-like positivity (Figure 4D).

## 4. Discussion

Cellular heterogeneity is increasingly recognized as an important prognostic feature of pediatric ependymomas. Single-cell and spatial transcriptomic studies have consistently shown that these tumors are not uniform but instead contain multiple cell populations with different biological programs [5,6,7]. Gillen et al. described undifferentiated progenitor-like cells, ependymal-like populations, astroglial-like cells, and hypoxia-associated mesenchymal states [5], while Gojo et al. reported a similar set of populations under different names, including neural stem-like, neuronal precursor-like, glial progenitor-like, and ependymal or astroependymal-like cells [6]. Despite the differences in terminology, both studies highlight that ependymomas harbor a mixture of differentiated and undifferentiated cells and that these programs have prognostic and therapeutic implications. This study demonstrates that such heterogeneity can also be appreciated using routine histology and basic immunohistochemistry, providing a bridge between molecular and morphological assessment. However, given the small, retrospectively assembled cohort and the limited representation of certain molecular subtypes, these observations should be regarded as preliminary and hypothesis-generating.

Low-cellularity areas showed strong diffuse GFAP positivity with a fibrillary background, resembling astrocytic or subependymal differentiation. These areas are morphologically very similar to subependymomas, which are benign GFAP-rich tumors, raising the idea of a morphological spectrum between subependymoma and ependymoma [8]. The WHO already recognizes mixed subependymoma–ependymoma tumors and grades them as ependymoma rather than subependymoma, reflecting the clinical importance of the higher-grade component [8]. Another possible interpretation of these GFAP-positive fibrillary areas is tanycytic differentiation. Tanycytes are specialized ependymal cells that extend GFAP-positive processes into the surrounding parenchyma [9]. This supports the idea that tanycytic or radial glial programs may also be represented within ependymomas. Since radial glia are considered progenitors in normal CNS development and have been implicated as a possible cell of origin in supratentorial ZFTA tumors [10], the finding of GFAP-positive fibrillary processes in ependymoma may reflect either astroglial, subependymal, tanycytic, or radial glial-like states. These possibilities remain difficult to resolve on morphology alone, but they illustrate the plasticity of these tumors.

EMA patterns also varied by region. In low-cellularity zones, classic dot-like or granular EMA positivity was present, consistent with ependymal differentiation. In high-cellularity zones, diffuse cytoplasmic EMA positivity was more frequent, particularly in ZFTA-fusion tumors, while GFAP expression was reduced. Numerous widespread dot-like EMA-positive cells have been suggested as a characteristic feature of *YAP1*-fusion ependymomas [11]. However, this dot-like pattern was also observed in the hypercellular regions of *ZFTA*-fusion ependymomas. In contrast, diffuse cytoplasmic EMA staining, as observed in these *ZFTA*-fusion tumors, has not been described in *YAP1*-fusion ependymomas.

Perinecrotic areas consistently showed increased diffuse cytoplasmic EMA positivity. One possible explanation is that these regions reflect stress-associated cellular states induced by hypoxia and inflammation. Hypoxia in these regions likely plays a central role: stabilization of HIF-1α and HIF-2α under low oxygen promotes the transcription of pro-inflammatory cytokines, including IL-6, IL-8, TNF-α, and TGF-β. Additionally, hypoxia can activate NF-κB signaling, further amplifying cytokine production. These locally elevated cytokines, together with oxidative stress and necrosis, may induce EMT-like changes or mesenchymal programs in tumor cells, consistent with observations in pediatric PFA ependymomas, where TGF-β1 and TNF-α drive mesenchymal gene expression via transcription factors such as NF-κB, AP-1, and MYC [12]. In gliomas, hypoxia has been shown to drive epithelial-to-mesenchymal-like transitions through HIF signaling [13]. In ST-ZFTA ependymomas, constitutive activation of NF-κB signaling by the *ZFTA*-*RELA* fusion may contribute to diffuse cytoplasmic EMA expression, potentially amplified by hypoxia- and necrosis-associated stress responses, providing a mechanistic link between the molecular driver and the observed immunophenotype. Mucin 1 (MUC1), the core antigen of EMA, is a stress-responsive protein upregulated by HIF-1α and NF-κB under hypoxic conditions [14], providing a potential molecular basis for the increased EMA expression in these regions. To date, however, this has not been systematically studied in pathology-based cohorts, so this interpretation remains speculative.

GFAP−/EMA− tumor cells were most common in hypercellular regions. These cells most likely correspond to the undifferentiated progenitor-like populations described in single-cell studies, which lack lineage markers but show malignant copy number alterations. Whether such cells represent radial glial precursors, tanycyte-like progenitors, or other stem-like states is unclear. The restricted use of GFAP and EMA in this study represents a limitation, as they alone cannot resolve these distinctions. Further investigation using additional lineage-specific immunostaining or molecular approaches could provide more precise characterization of these undifferentiated tumor populations.

The present results also highlight important subtype-specific differences. ST-ZFTA tumors were uniformly hypercellular and often showed strong diffuse EMA immunostaining. In contrast, PFA tumors were more variable, often displaying subependymoma-like GFAP-rich low-cellularity areas and a broader range of EMA profiles. These findings are consistent with prior work by Gödicke et al., who showed that cell-dense PFA regions are enriched for chromosomal alterations such as 1q gain and 6q loss and correlate with poor outcome. The current study did not include molecular testing, but the morphological parallels support the idea that cell density and heterogeneity are not random but reflect underlying biology. At the same time, the number of cases in each molecular subtype, particularly the single PFB case and the absence of YAP1-fusion supratentorial ependymomas, is small and introduces an unavoidable selection bias. As a result, the subtype-specific observations reported here should be interpreted with caution and validated in larger, more representative cohorts before they are used in routine practice.

The presence of astrocytic-appearing cells raises the question of whether they are reactive or neoplastic. Spatial studies indicate that most are neoplastic [7]. In this study, both reactive-looking astrocytes and neoplastic astrocyte-like tumor cells were observed, suggesting that tumor cells can adopt multiple astrocytic morphologies.

Groh et al. demonstrated that ependymal cells exhibit a reactive transcriptomic profile similar to that of astrocytes in response to neuroinflammation, suggesting that ependymal cells possess plasticity that allows them to adopt astrocytic morphologies under certain conditions [15]. Recent studies further highlight the clinical relevance of infiltrative components in ependymomas. Their analysis of 229 intracranial ependymomas demonstrated that local infiltration is an independent prognostic factor [16]. Another PFA ependymoma exhibited widespread infiltration and had a worse prognosis, resembling astrocytoma, thereby blurring the traditional diagnostic boundaries between the two entities [17]. Additionally, some ependymomas show significant OLIG2 immunostaining [18], consistent with transdifferentiation into glial-precursor-like cells in a subset of tumor cells.

These findings underscore the complexity of ependymoma pathology and the challenges in distinguishing between reactive and neoplastic astrocytic features. The ability of ependymal cells to transdifferentiate into infiltrative glial cells, such as astrocytes, may have significant implications for prognosis, as local infiltration is a recognized negative prognostic factor. This phenomenon also complicates the grading of high-grade gliomas with infiltrative components, raising the question of whether such cases should be classified as high-grade ependymomas with astrocytic differentiation or as glioblastomas with ependymal-like areas. In this study, we highlighted the astrocytic GFAP staining quality of specific tumor cells, and in one case, an infiltrative glioma-like pattern developed. This observation suggests that ependymomas may exhibit greater histopathological and molecular heterogeneity than previously recognized, including the potential for transdifferentiation into infiltrative glial cell types. Further research utilizing advanced molecular techniques and lineage-specific immunostaining is essential to elucidate the mechanisms underlying this plasticity and its clinical implications.

A limitation of this study is that only slides with available GFAP and EMA immunostaining were evaluated, as the analysis was performed using digital archival images. Consequently, certain tumor regions without immunostaining could not be assessed, which may have led to underestimation of cellular heterogeneity in some cases. The small cohort size, semi-quantitative scoring, and reliance on archival digital slides also limit the study. While GFAP and EMA provided valuable information on differentiation patterns, additional markers such as FOXJ1, SOX9, or OLIG2 could better distinguish ependymal from glial progenitor differentiation. Future studies incorporating spatial transcriptomics, multiplex immunostaining, and advanced digital pathology will be essential to confirm whether perinecrotic GFAP and EMA upregulation reflect EMT-like or stress-associated programs. Overall, this work should be viewed as a preliminary, proof-of-principle study showing that aspects of the cellular heterogeneity described by recent single-cell and spatial transcriptomic studies can be recognized on routine slides with basic immunostains. It does not aim to, and cannot, supplant molecular classification, but rather to complement it and provide a framework for future, larger studies that more rigorously link histological patterns, molecular subtypes, and clinical outcome.

## 5. Conclusions

This study demonstrates that cellular heterogeneity in pediatric ependymomas can be appreciated using routine histology and basic immunohistochemistry. Low-cellularity regions frequently exhibit GFAP-rich fibrillary differentiation, which may reflect astroglial, subependymal, tanycytic, or radial glial-like programs. In contrast, hypercellular zones are enriched for EMA-positive ependymal cells and GFAP/EMA-negative progenitor-like populations. Perinecrotic regions display distinctive profiles suggestive of stress-associated differentiation.

These findings provide proof of principle that heterogeneity can be evaluated histologically, even with simple immunostains, and highlight the advantage of pathological assessment in surveying large tumor areas and spatial relationships between distinct cellular populations, including infiltrative components. At the same time, the small sample size and incomplete coverage of all molecular subtypes mean that these observations cannot be used to replace molecular testing and should not be interpreted as formal diagnostic criteria.

Future work utilizing additional immunostaining panels and larger cohorts could improve the accuracy of histological assessment and prognostic stratification, particularly in settings where molecular testing is not readily available. By combining histopathology with spatial and molecular approaches, more precise predictions of tumor behavior and clinical outcomes may be achievable.

## Data Availability

All relevant data files are available from the corresponding author (MA) upon request.
